# Surgical Application of Galvanism

**Published:** 1859-01

**Authors:** Brown Sequard


### ARTICLE VIII.

*Surgical Application of Galvanism.* By DR. BROWN SEQUARD. Translated for the American Journal of Dental Science.

THE Americans have resolved to combat pain by all possible means. After having discovered etherization, they propose now to employ a procedure, which, if not destined to be useful in a great number of surgical operations, and if not so powerful as chloroform, has nevertheless, this advantage, that it will not kill any one. In this case, as in the inhalation of ether, dentists have made the discovery, and for the extraction of diseased teeth. It is the application of a galvanic current through the part upon which the operation is to be performed. Committees appointed by learned societies in Philadelphia and Baltimore, have made very favorable reports upon the utility of employing a galvanic current applied to the gums (!) during the extraction of diseased teeth. The commission appointed by the Franklin Institute of Philadelphia, found in several hundred cases of extraction of teeth, that, in the majority of cases, the patients were free of pain. The dentists of England, and a dentist of Paris have made use of this process; but this is not all that can be done with galvanism; a surgeon of the hospital of the University of London, Mr. Marshall, has attempted to render other parts of the body than the gums, insensible by means of the galvanic current. The medical journals of London report that nine operations have been performed, consisting of the opening of abscesses and boils, the extirpation of a necrosed bone, and the removal of a fatty tumor. The general effect produced by galvanism has consisted in a diminution of the pain by rendering it less acute.

I.—*Account of* Dr. Brochin, *Editor of the Gazette des Hopitaux.*

A new method of producing local anæsthesia, applied to the extraction of teeth, has created some noise. Several surgeons of the hospital have been surprised by this invention, which has been brought forward by honorable men perfectly competent to judge; they have experimented, and the results of their experiences have been brought before the academy. These results, it may be well to add, have not answered fully our expectations after the very precise and distinct communications which have been made both to the academy and in the journals. There have appeared among them contradictions little calculated to produce conviction. Thus, after a certain number of experiments by M. Robert, at the Hotel Dieu, and of which he gave an account to his colleagues, the current produced no effect in two cases, in many cases it remained doubtful, and only four applications of the new method were without pain. From M. Nelaton we learn that similar experiments had been made for him by M. Preterre, and that the success was complete. M. Nelaton being absent, M. Moreau assisted, and has made known the results. Finally, M. Velpeau states, that after several trials at incisions, using an analogous method, he has not met with any thing satisfactory. And so it seems, that up to the present time, these attempts have not been sufficiently numerous to authorise a definite conclusion as to the success or failure of the method, and have only left doubts on the minds of the experimenters. Must we ask with M. Velpeau "is there any difference between the American teeth and ours?" Not having witnessed the experiments, we would rather allow our doubts to weigh upon the perfect identity of the conditions of the experiments until new and similar ones show us that there has been inexperience on one side or illusion on the other.

### II.—*Account of* M. H. DE CARTELNAU, *Editor of the Moniteur des Hopitaux.*

THIS is not the first time that we have had occasion to felicitate M. Robert upon the zeal he has evinced in the progress of the art of surgery, upon suggesting them himself, upon extending and judging them; but repetitions of this kind do not generally displease—especially those who are the objects; we will, therefore, to-day, renew our felicitations to M. Robert. M. Robert has desired to know if there is any truth in the discovery of the American dentist, Mr. J. B. Francis. In the hands of M. Robert the results of the discovery have not been so good as in those of his trans-atlantic confrere. Still a method which gives four successes in ten trials is not to be despised. M. Robert is not satisfied in applying electricity to the extraction of teeth; he wishes to discover if something cannot be made of the discovery for surgery in general. But here he has been completely disappointed.

M. Velpeau is also a promoter of progress and does not wish to give up his place to others; he has taken upon himself, equally with others, the work of judging the new method, followed by one of his most distinguished pupils, who has passed to rank with masters, M. Follin. Once in four times only has M. Velpeau been successful in the use of galvanic anæsthesia; but he has only experimented in cases of surgery proper, so that they do not affect the doubtful question concerning its application in the dental art. M. Nelaton pretends also, or at least has the right to pretend—for generally he has more right than pretension—that he is not behindhand, and if M. Moreau is to be believed, and in two cases of extraction of teeth, M. Nelaton was twice successful. This is truly something. For our own part we have assisted with several physicians, in making numerous experiments for the promotion of these communications; we have not counted the cases, but we are able, nevertheless, to say that in the great majority of cases the success has been such that the patients said they experienced no pain.

To sum up, the method of Mr. Francis, restricted entirely to the extraction of the teeth, seems to be a success. We do not believe, in short, that the emotion enters in the place of something in the absence of pain,* as M. Robert appears to have thought in making allusion to the practice of Dupuytren. The emotion is caused more by the fear of the operation than by the operation itself, and if the patients do not suffer in some cases, and suffer in others, it is assuredly to the means employed that the merit is attributable.

[*L'Art Dentaire.*

### ARTICLE IX.

### *Dentistry among the Ancients.*

* Que l'emotion entre pour quelque chose dans l'absence de doulear.

